# Serum inflammatory biomarker profiles across the Alzheimer's disease spectrum in the Bio‐Hermes cohort

**DOI:** 10.1002/alz70861_108502

**Published:** 2025-12-23

**Authors:** Zain Hussain, Dominic Ng, Samuel Leighton, Fani Deligianni, Craig Ritchie, Jonathan Cavanagh

**Affiliations:** ^1^ University of Glasgow, Glasgow, Glasgow UK; ^2^ Scottish Brain Sciences, Edinburgh, Scotland UK; ^3^ University of Edinburgh, Edinburgh, Scotland UK; ^4^ University of Glasgow, Glasgow, Scotland UK; ^5^ University of St Andrews, St Andrews, Scotland UK

## Abstract

**Background:**

Neuroinflammation is an important contributor to Alzheimer’s disease (AD) pathogenesis. However, it is unclear whether inflammation drives, precedes, or occurs concurrently with AD pathology, and how inflammatory markers relate to established AD biomarkers. This study used the Bio‐Hermes cohort to investigate associations between a comprehensive serum inflammatory panel, AD biomarkers, and key clinical/biological factors.

**Method:**

Cross‐sectional data from 964 participants in the Bio‐Hermes study, comprising cognitively normal (CN=404), mild cognitive impairment (MCI=302), and mild AD dementia (AD=258) groups, were analyzed. Serum concentrations of 32 cytokines, NfL, GFAP, and AD biomarkers (Aβ40, Aβ42, Aβ42/Aβ40, total tau, *p* ‐tau181, and *p* ‐tau217) were included. Age/sex‐adjusted ANCOVA compared cytokine, NfL, and GFAP levels across cognitive groups, stratified by amyloid and APOEε4 status (Tukey's HSD, FDR *p* <0.05). Pearson correlations assessed relationships between significant cytokines and AD plasma biomarkers across cognitive groups. Linear regressions assessed associations with clinical scores (MMSE, FAQ, RAVLT, GDS), adjusted for age, sex, education, and APOEε4.

**Result:**

Significant differences across cognitive groups were found for NfL, GFAP, and 24 cytokines. NfL, GFAP, and Eotaxin‐2/CCL24 showed stepwise increases (CN<MCI<AD). Seventeen cytokines (including Eotaxin/CCL11, SCF, IL‐1β, TNF‐RII) were elevated in AD versus both CN and MCI; IL‐7 was lower. IL‐2R was elevated in MCI and AD vs CN. Four cytokines (IFN‐γ, IL‐18, MCP‐2/CCL8, MIP‐1α/CCL3) were elevated in AD vs CN only. Most group differences persisted within amyloid‐positive individuals. APOEε4 carriers had lower GFAP but higher IL‐15, IL‐20, and MCP‐2/CCL8. Higher NfL, GFAP, IL‐17A, IL‐7 associated with lower MMSE in mild AD (IL‐18 in MCI); higher Eotaxin‐2 and IL‐2R associated with better RAVLT scores, and higher IL‐17A with lower RAVLT scores (*p* <0.05).

**Conclusion:**

Serum inflammatory alterations, including 24 cytokines, strongly associate with cognitive status in Bio‐Hermes, largely independent of amyloid pathology. Distinct inflammatory markers associate with APOEε4 genotype. Differences in biomarker correlation patterns and clinical associations highlight immune dysregulation across the AD spectrum. These findings reinforce inflammation’s role in AD pathophysiology, suggesting avenues for biomarker development and immunomodulatory therapies, warranting longitudinal investigation.

